# Information Architecture of Web-Based Interventions to Improve Health Outcomes: Systematic Review

**DOI:** 10.2196/jmir.7867

**Published:** 2018-03-21

**Authors:** Jillian Pugatch, Emily Grenen, Stacy Surla, Mary Schwarz, Heather Cole-Lewis

**Affiliations:** ^1^ ICF Rockville, MD United States; ^2^ ICF Fairfax, VA United States; ^3^ Johnson & Johnson Health and Wellness Solutions, Inc New Brunswick, NJ United States

**Keywords:** information architecture, systematic review, health outcomes, behavior change, health behavior

## Abstract

**Background:**

The rise in usage of and access to new technologies in recent years has led to a growth in digital health behavior change interventions. As the shift to digital platforms continues to grow, it is increasingly important to consider how the field of information architecture (IA) can inform the development of digital health interventions. IA is the way in which digital content is organized and displayed, which strongly impacts users’ ability to find and use content. While many information architecture best practices exist, there is a lack of empirical evidence on the role it plays in influencing behavior change and health outcomes.

**Objective:**

Our aim was to conduct a systematic review synthesizing the existing literature on website information architecture and its effect on health outcomes, behavioral outcomes, and website engagement.

**Methods:**

To identify all existing information architecture and health behavior literature, we searched articles published in English in the following databases (no date restrictions imposed): ACM Digital Library, CINAHL, Cochrane Library, Google Scholar, Ebsco, and PubMed. The search terms used included information terms (eg, information architecture, interaction design, persuasive design), behavior terms (eg, health behavior, behavioral intervention, ehealth), and health terms (eg, smoking, physical activity, diabetes). The search results were reviewed to determine if they met the inclusion and exclusion criteria created to identify empirical research that studied the effect of IA on health outcomes, behavioral outcomes, or website engagement. Articles that met inclusion criteria were assessed for study quality. Then, data from the articles were extracted using a priori categories established by 3 reviewers. However, the limited health outcome data gathered from the studies precluded a meta-analysis.

**Results:**

The initial literature search yielded 685 results, which was narrowed down to three publications that examined the effect of information architecture on health outcomes, behavioral outcomes, or website engagement. One publication studied the isolated impact of information architecture on outcomes of interest (ie, website use and engagement; health-related knowledge, attitudes, and beliefs; and health behaviors), while the other two publications studied the impact of information architecture, website features (eg, interactivity, email prompts, and forums), and tailored content on these outcomes. The paper that investigated IA exclusively found that a tunnel IA improved site engagement and behavior knowledge, but it decreased users’ perceived efficiency. The first study that did not isolate IA found that the enhanced site condition improved site usage but not the amount of content viewed. The second study that did not isolate IA found that a tailored site condition improved site usage, behavior knowledge, and some behavior outcomes.

**Conclusions:**

No clear conclusion can be made about the relationship between IA and health outcomes, given limited evidence in the peer-reviewed literature connecting IA to behavioral outcomes and website engagement. Only one study reviewed solely manipulated IA, and we therefore recommend improving the scientific evidence base such that additional empirical studies investigate the impact of IA in isolation. Moreover, information from the gray literature and expert opinion might be identified and added to the evidence base, in order to lay the groundwork for hypothesis generation to improve empirical evidence on information architecture and health and behavior outcomes.

## Introduction

With the rise of new technology and digitization of our physical information environments, it is important to understand the role of digital information organization on user outcomes. This may be particularly important for the information architecture (IA) of Web content [1]. While no one definition of IA exists, it generally encompasses the organization of digital information, the labeling of information, and the navigation and search capabilities within a digital information space. The goal of IA is to build digital sites that enhance the user experience—in particular, the user’s ability to find and use content [2].

IA is vital to website development. In commercial settings, good IA can enhance the ability of employees and customers to find information and decrease costs of Web redesign and maintenance [2]. However, IA is less often discussed in the context of digital spaces for behavior change and health outcomes. Moreover, IA best practices for commercial settings may not translate to health-related ones, where user needs are entirely different [2]. A user seeking information that is factual, concrete, and that they know exists (eg, the price of a new computer or the weekend forecast) will benefit from different site architecture than the user who wants to quit smoking or manage weight loss. In the latter scenarios, the information sought may be complicated and unfamiliar; the user may not even know exactly what information they should be seeking. Thus, while many IA recommendations exist, there is still a lack of empirical evidence for the role that IA plays in Web-based health behavior interventions.

Digital health interventions that mention IA primarily focus on navigation systems [3-5]. Generally, navigation systems concern the relationships among information or content at different levels—such as Web pages or sections. Structures can be hierarchical (top-down approach, with broader subjects encompassing smaller ones), matrix (movement along multiple dimensions), organic (free movement or exploration), or tunnel (sequential or linear organization) [6].

Many experts in the field recommend and implement a tunnel (or tunnel hybrid) design for behavior change websites. A recent systematic review of Web-based health intervention studies showed that tunneling structures were used in 90% of interventions reviewed. Of the interventions reviewed, all of those with a mental health focus used tunnel designs [7]. Users of websites with a tunnel design navigate in a sequential fashion to optimize the ordering of information and maximize the effectiveness of the site, in much the same way that one would read a novel or watch a television series from start to finish [1]. An example of tunnel design might be an online app that takes the user through a series of steps in a sequential order (eg, the app for health insurance on the American HealthCare.gov website), or a site with an e-learning module where lessons are presented in a predetermined order [1,8]. A tunnel experience is less likely to overwhelm users with information and options; it simplifies information consumption by defining what the user sees and when. In addition, tunnel design has the capacity to provide tailored “remedial” loops for users who do not pass certain knowledge test “check-points” or assessments [1]. In general, this type of feedback and reinforcement personalizes the experience and helps the individual progress through an intervention program. Evidence shows that personalized Web interventions are more efficacious in behavior change [1,9].

A hybrid design that includes elements of tunnel design provides an opportunity to give users more structure and guidance while also allowing a user to break free from a “locked” information structure if they so choose [1]. A website with a hybrid design might, for example, offer the user a table of contents that allows that individual to view website pages in any order. However, this same site might also include links within certain pages that direct users to a logical next step, thereby providing an element of tunnel design (eg, the National Institute of Justice’s Laboratory Safety Training website) [10]. A hybrid tunnel design has the capacity to offer the user various ways of consuming the information, which may incentivize the user to take a more active role in their learning experience rather than simply turning pages, which is a risk with tunnel-only designs [1]. Hybrid design may also reduce attrition rate of a full tunnel design, as it does not deter individuals who may find the tunnel design too inflexible [1].

Conversely, free-form matrix—also known as organic—and hierarchical designs are less suitable for users unfamiliar with the content area (as is often the case for users of behavior change sites) because the freedom to explore information may make it difficult to navigate [1]. Additionally, these designs can make it more challenging for users to retrace their information search in order to review something previously seen [1].

Despite the aforementioned recommendations and the attention IA has received in the commercial sector, IA is largely a missed opportunity in the health behavior field. Most digital health intervention research describes the studies but fails to address the actual features of the Web tools being used, such as their IA [11-13]. Yet, understanding and implementing IA designs that best promote behavior change may be a simple and sustainable way to significantly improve the efficacy of digital interventions.

Thus, this review synthesizes the existing literature on website IA in the context of Web-based health interventions. We examine whether manipulating the information architecture of Web-based health interventions influences website use, health behaviors, and outcomes.

## Methods

### Inclusion and Exclusion Criteria

Articles were considered eligible if they met all inclusion criteria. In addition to being peer-reviewed and published in English, studies were included if they were (1) a randomized controlled trial (RCT), (2) an assessment of the effect of one type of IA compared to any other type of IA, (3) an intervention delivered in a Web-based setting, and (4) included either a primary health outcome measure (eg, disease status) or a secondary, proximal health outcome measure including change in knowledge, attitudes, or beliefs (eg, hepatitis knowledge) relating to the target health behavior, behavior change (eg, number of cigarettes smoked), website engagement (eg, number of pages visited), or attitudes towards the website (eg, perceived user control). No date restrictions were imposed. Interventions could address any health issue (eg, mental health, chronic conditions, and communicable diseases). Studies were included only if interventions were Web-based; interventions that focused on mobile apps or games, for example, were excluded.

### Search Strategy

Literature searches were conducted on March 30, 2015. The following electronic databases were searched: ACM Digital Library, CINAHL, Cochrane Library, Google Scholar, Ebsco, and PubMed. The search terms used included information terms (eg, information architecture, interaction design, persuasive design), behavior terms (eg, health behavior, behavioral intervention, ehealth), and health terms (eg, smoking, physical activity, diabetes) (see Multimedia Appendix 1).

Eligibility assessment was performed independently by 2 reviewers. Disagreements between reviewers were resolved by consensus that included a third reviewer.

### Data Extraction and Synthesis

A data extraction form was developed based on a priori categories established by 3 reviewers. Due to the small number of articles included in the review, this form was piloted on the three publications included in the systematic review.

Information was extracted from each included study on (1) characteristics of participants (including age, disease/behavior status), (2) type of intervention, (3) types of information architecture manipulated, (4) duration of the study, (5) website engagement outcomes, (6) knowledge, attitudes, and beliefs outcomes, and (7) health outcomes.

To determine the validity of eligible randomized trials, the pair of reviewers used the Cochrane Collaboration tool for assessing risk of bias in individual studies [14]. Disagreements in quality assessments were resolved by discussion between the 2 reviewers.

## Results

### Findings

**Figure 1:**
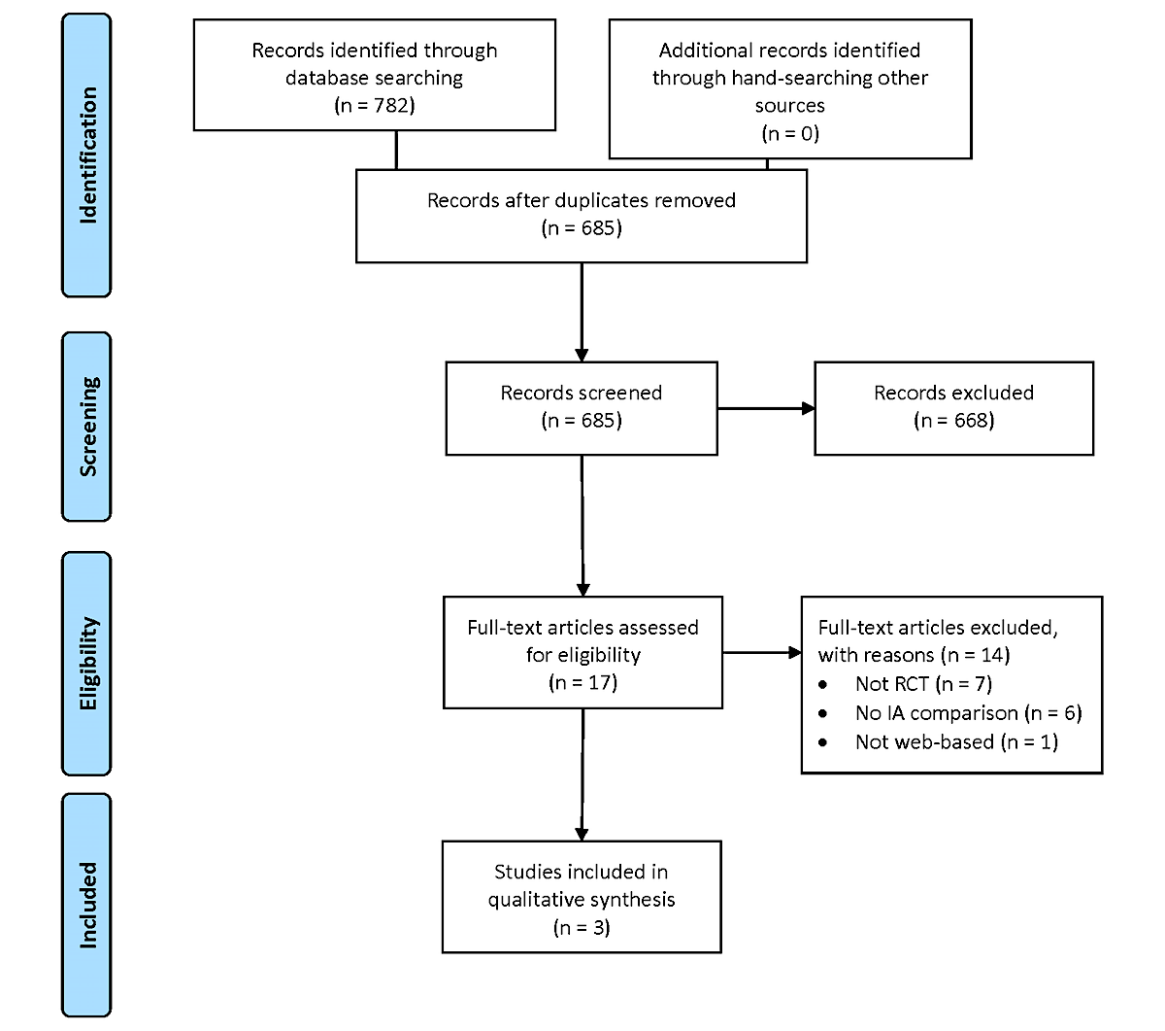
Preferred Reporting Items for Systematic Reviews and Meta-Analyses flow diagram. RCT: randomized controlled trial; IA: information architecture.

Figure 1 illustrates the number of studies identified, screened, and included in this literature review [15]. The database literature search produced 782 citations. After duplicate citations were removed and the abstracts were reviewed, 17 citations met the inclusion criteria. The full text of these remaining citations were reviewed, and 14 were excluded because of study design (non-RCTs), a lack of IA manipulation, a lack of primary or secondary health outcome measures, or because they studied a non‒Web-based platform. Three articles were included in this systematic review [16-18].

### Risk of Bias Assessment

The risk of bias criteria and outcomes are described in Table 1. Overall, risk of bias for all studies was low. Two studies failed to clearly report their method of random sequence generation [17,18], and one failed to report methods of allocation concealment and blinding of participants and personnel [17].

### Study Characteristics

A summary of notable study characteristics is reported in Table 2. Sample sizes ranged from 561 [16] to 2523 [17] participants. One study was conducted in the United States [17], one in the Netherlands [18], and the third in Germany [16]. All studies were published in English.

### Participants

Although all three studies lost participants to follow-up, only Weymann et al noted selective dropout, which occurred among participants with chronic lower back pain [16]. Those in the tailored condition were younger (mean 48.0, SD 12.9) and had higher education defined by having more than 10 years of education (119/190, 62.6%) than those in the control (age: mean 52.0, SD 12.7, *P*=.015; education: 94/188, 50.0%, *P*=.021). This study conducted intention-to-treat (ITT) and available cases (AC) analyses in order to determine the extent to which selection bias may have impacted the results.

### Intervention Characteristics and Outcomes

The manipulation solely of IA was studied in only one publication [18], making it difficult to attribute the other two studies’ results to the difference in IA. The two other studies manipulated website features (eg, interactivity, email prompts, and forums) and tailored content in addition to IA. All studies assessed some form of tunnel architecture against an organic architecture. Outcomes assessed included number of pages visited, time on site, website attrition, knowledge, perceived user control, perceived control, decisional conflict, patient empowerment, preparation for decision making, and change in knowledge.

Given that only one of the three publications assessed the isolated effect of IA, intervention characteristics and effect of IA on outcomes of interest are presented by study and categorized by whether the effect of IA was isolated. Table 3 includes more details regarding the studies’ designs, results, and conclusions.

### Interventions Assessing the Isolated Effect of Information Architecture

#### Crutzen et al Intervention Characteristics

The Crutzen et al study involved two versions of a website with different information architectures and a no-website control group [18]. This publication assessed tunnel versus organic architecture.

One intervention group used a website about hepatitis with tunnel design. The pages on this site could be viewed only in a predetermined order and pages could not be skipped. The second group visited a freedom of choice (organic) site with identical content and the same number of pages as the tunnel version, but users had the ability to skip pages.

#### Crutzen et al Outcomes

Participants in the tunnel condition visited more pages (mean 11.4) compared to those in the freedom of choice condition (mean 7.4, *P*<.001). Users in the tunnel condition also spent more time on the site the than freedom of choice users (3:50 minutes compared to 2:38 minutes; *F*_1,452_=6.32, *P*=.01).

Less user control had a negative effect on perceived website efficiency (*P*<.01), but a positive effect on knowledge gained (*P*<.001). Participants in the tunnel group scored higher on hepatitis knowledge compared with the freedom of choice group (*P*<.001).

### Interventions Assessing the Non-Isolated Effect of Information Architecture

#### Danaher et al Intervention Characteristics

Danaher et al exposed smokeless tobacco users to a Basic and an Enhanced website for smokeless tobacco cessation called Chewfree.com [17]. The article assessed hybrid tunnel versus organic architecture. The Enhanced condition offered a tailored and interactive Web-based program that included text-based information, video-based testimonials, printable resources, interactive activities, annotated links to other website resources, and two Web forums. The Enhanced site used five navigational pages (one of which used a hybrid design that incorporated tunneling). The Basic condition represented a subset of the content presented in the Enhanced condition and included text-based content using four navigational pages. It also offered a printable self-help smokeless tobacco cessation booklet, printable cessation resource, and annotated links to other recommended websites for tobacco cessation.

**Table 1:** Risk of bias assessment^a^ for individual studies.

Criteria	Weymann et al [16]		Danaher et al [17]		Crutzen et al [18]	
	Risk of bias	Support for judgment	Risk of bias	Support for judgment	Risk of bias	Support for judgment
Random sequence generation	Low	Simple randomization of participants performed by a software program	Unclear	No description of the methodology to generate allocation sequence	Unclear	No description of the methodology to generate allocation sequence
Allocation concealment	Low	Randomization software assured the concealment of allocation.	Unclear	No description of methods used to generate intervention or control allocations. However, given the study took place online, it is unlikely that participants would be aware of allocation.	Low	No description of methods used to generate intervention or control allocations. However, participants were not informed about the existence of these 3 groups or that the study focus was on website use.
Blinding of participants and personnel	Low	Participants were aware that there were intervention and control groups, but blinded to their assignment. However, authors stated “it might be possible that participants identified the intervention group due to the unusual dialogue-based delivery format used in the intervention group.” Due to software-automated allocation, personnel remained blinded.	Unclear	No description of participant or researcher blinding. However, given study took place online, it is unlikely that either participants or researchers would have been aware of the intervention and control allocations.	Low	No description of participant or researcher blinding. However, given the study took place online, it is unlikely that either participants or researchers would have been aware of intervention and control allocations.
Blinding of outcomes assessment	Low	Outcomes all collected via self-reported questionnaires.	Low	Website use and engagement comprised all outcomes measured, which were calculated via an automated computer program.	Low	All outcomes were collected via a computer server (website use) or via self-reported questionnaires completed online.
Incomplete outcome data	Medium	Although authors stated that “attrition was comparatively low for an online trial,” they found evidence for selective dropout between the control and intervention conditions.	Low	Only about 6% of participants were not included in the analyses. This included participants who never visited their assigned website or returned only to complete online assessments.	Low	Relatively small dropout rate between pre-test and follow-up, and authors reported that there was neither selective dropout nor a difference in dropout between conditions.
Selective reporting	Low	Data for all outcomes described in the study protocols^b,c^ were reported.	Low	Data from all outcomes indicated in the Methods section reported in the Results section.	Low	Data from all outcomes indicated in the Methods section reported in the Results section.
Other bias	Low	None identified.	Low	None identified.	Low	None identified.

^a^Risk of bias was categorized as low, medium, or high based on whether reviewers thought the methods or descriptions indicated a low, medium, or high risk. “Unclear” risk of biases was noted for studies that lacked a description of that domain.

^b^[19].

^c^[20].

#### Danaher et al Outcomes

Specific tunnel elements and IA were not isolated in this intervention. The Enhanced site generated more usage. Participants in the enhanced condition made more visits and spent more time on the site than participants in the Basic condition (*P*<.001). Users in the Enhanced condition continued to use the site for more days than Basic website users (*P*<.001). Interestingly, more cessation content was visited in the Basic condition, though the study authors note that this could be due to the same content being difficult to find in the Enhanced site.

#### Weymann et al Intervention Characteristics

Weymann et al compared a tailored and interactive site with some tunneling elements to a control site without tunneling [16]. The study assessed dialogue-based and tunnel versus organic architecture. There were intervention and control sites for people with type 2 diabetes and chronic lower back pain for a total of four conditions. The look of the websites (colors, font, figures, and pictures) was identical in all conditions, and participants could view sites as often as they wished.

In the tailored conditions, the delivery format was a dialogue-based, tunnel design. The dialogue aspect of the design attempts to imitate a conversation with a health professional. Various check-points assessed user knowledge and attitude toward a topic, and content was then modified according to their answer. Users were given limited control over the sequence in which they viewed content—although they were permitted to pick from one of several options at the end of each text passage. On the control websites, the content was not tailored and was not presented in a dialogue format. In contrast to the tailored, interactive version, the control website users were given freedom to view content in any order by selecting topics from a menu.

#### Weymann et al Outcomes

Analyses and findings of this study did not explore IA specifically. In this study, the tailored and tunnel conditions spent more time on the site (51.16 minutes) than the control groups (37.6 minutes) (*P*<.001). Results for the other outcomes are as follows:

- Knowledge after the first visit ‒ ITT: no significant difference (*P*=.53); AC: tailored group had significantly more knowledge (*P*=.02) than control
- Patient empowerment ‒ ITT: no significant difference; AC: tailored group had better emotional well-being (a subscale of empowerment) than control (*P*=.009)
- Decisional conflict ‒ disease main effect for ITT and AC
- Preparation for decision making ‒ ITT: no significant difference; AC: disease main effect (*P*=.02)

Content tailoring and interactivity may increase knowledge and reduce health-related negative effects in persons who use interactive health communication apps.

**Table 2:** Characteristics of included studies.

Study	Intervention arms	Population	Sample size	Health concern	Outcome measures^a^
Weymann et al [16]	Tunnel condition: Tunnel design and tailored content	Adults in Germany with access to internet and sufficient computer/internet literacy. Participants had either a self-reported diagnosis of type 2 diabetes or chronic low back pain.	Baseline (n=561): Tunnel condition n=283; Control condition n=278	Type 2 diabetes; Chronic lower back pain	(1) Time on website, (2) Knowledge after first website visit, (3) Decisional conflict after 1st website visit, (4) Preparation for decision making after 1st website visit, (5) Patient empowerment at 3-month follow-up
	Control condition: Free-form navigation website with untailored content not presented in a dialogue format		Follow-up (n=295): Tunnel condition n=146; Control condition n=149		
Danaher et al [17]	Enhanced condition: Hybrid tunnel design website with interactive, tailored, rich media	Adult smokeless tobacco users in the United States.	Baseline (n=2523): Enhanced condition n=1260; Control condition n=1263	Smokeless tobacco use	(1) Website visits at T1, T2, and T3, (2) Time on website at T1, T2, and T3, (3) Website attrition from T1-T3
	Control condition: Static, text-based website with free navigation to all content		Follow-up (n=2375): Enhanced condition n=1200; Control condition n=1175		
Crutzen et al [18]	Tunnel condition: Website with tunnel design and less user control	Adult internet users in the Netherlands.	Baseline (n=668): Tunnel condition: n=226; Free-form condition: n=228; Control: n=214	Hepatitis	(1) Time on website at T0, (2) Number of pages viewed at T0, (3) Perceived user control at T1, (4) User perceptions at T1, (4) Change in hepatitis knowledge from T0-T2
	Free-form condition: Freedom of choice design where users had ability to skip pages		Follow-up (n=571): Tunnel condition: n=200; Free-form condition: n=193; Control: n=178		
	Control condition: No exposure to website				

^a^T0=baseline, T1=time 1, T2=time 2, and T3=time 3, when user data were collected.

**Table 3:** Results and conclusions of included studies.

Author	Data collection points	Website use results	Knowledge, attitudes, beliefs results	Conclusion
Weymann et al [16]	T1: Immediately after 1st website visit, T2: 3-month follow-up	Time on website: Tunnel condition mean 51.2 min; Control condition mean 37.6 min (*P*<.001)	Knowledge after 1st visit: ITT^a^ analysis=Tailored condition mean 77.9; Control condition mean 76.3 (*P*=.53). AC^b^ analysis=Tailored condition mean 79.1; Control condition mean 75.2 (*P*=.02)	Participants spent more time with tunnel site than the control. In the ITT analyses, this did not result in more knowledge or empowerment. Sensitivity analyses (AC) showed that participants in tunnel condition displayed more knowledge and emotional well-being. However, on other measures of patient empowerment, there was no difference between the 2 conditions.
			Decisional conflict after 1st visit: No significant intervention main effects for AC or ITT analyses.	
			Preparation for decision making after 1st visit: No significant intervention main effects for AC or ITT analyses.	
			Patient empowerment at 3-month follow-up: ITT analysis=No significant intervention main effect or interaction. AC analysis=Intervention main effect for Emotional Well-being (subscale of patient empowerment). Tailored condition mean 68.5; Control condition mean 60.0 (*P*=.009).	
Danaher et al [17]	T1: 6 weeks after enrollment, T2: 3 months after enrollment, T3: 6 months after enrollment	Website visits: Enhanced condition made more visits (*z*=-16.64, *P*<.001, 2-tailed).	N/A^c^	Study suggests that hybrid tunnel IA may encourage higher participant engagement with website content than free-form IA. Engagement measures are important in understanding program effectiveness. However, the study is limited in that it does not directly measure behavioral outcomes.
		Time on website: Enhanced condition spent more time viewing website content (*z*=-17.63, *P*<.001, 2-tailed).		
		Website attrition: Enhanced condition showed slower attrition (*P*<.001 for both log-rank and Breslow tests).		
Crutzen et al [18]	T0: Pretest, T1: Immediately after viewing website, T2: 1 week after viewing website	Time on website: Tunnel condition mean 3:50 min; Free-form condition mean 2:38 min (*F*_1_,_452_=6.32, *P*=.01).	Perceived control: Free-form condition higher mean 5.2; Tunnel condition mean 3.9 (*F*_1,452_=134.32, I<.001)	IA that provides less choice may improve intervention engagement and disease knowledge, which may benefit health behavior outcomes. However, user perceptions of efficiency may be compromised by restricting user choice.
		Number of pages visited: Tunnel condition mean 11.4 pages; Free-form condition mean 7.4 pages (*F*_1,452_=171.49, *P*<.001).	Change in hepatitis knowledge: Tunnel condition pretest mean 5.0, posttest mean 8.2; Free-form pretest mean 5.4, posttest mean 7.2; Control condition pretest mean 5.4, posttest mean 5.6 (*F*_2,567_=47.24, *P*<.001). All pairwise comparisons significant (*P*<.001).	

^a^ITT: intention-to-treat.

^b^AC: available cases.

^c^N/A: not applicable.

## Discussion

### Principal Findings

Given the limited body of evidence connecting IA to behavioral outcomes and website engagement, no clear conclusions can be made about the relationship between IA and health outcomes. Moreover, several weaknesses in the design of the studies identified make it challenging to generalize results. Only one of the articles, for example, explicitly and empirically manipulated IA by itself [18]. The other studies included other manipulations to website features and tailoring, making it difficult to attribute the results to the difference in IA. Loss to follow-up also makes it difficult to determine whether outcomes resulted from the intervention itself or simply bias [16]. Future empirical research on IA necessitates more robust study designs that isolate the effect of IA and minimize loss to follow-up. Adopting a more nuanced study design approach may even allow researchers to isolate IA while testing other features of an intervention in an RCT. For example, the Sequential Multiple Assignment Randomized Trials (SMART) design for adaptive interventions—in which participants move through multiple stages of an intervention and get reassigned to several intervention options—might offer an opportunity to test IA features tailored to particular users depending on their behaviors and needs within the context of a larger intervention trial [21].

The publications in this review did not assess health outcomes—instead they focused on more proximal outcomes such as behavior change and website engagement. It is generally accepted that some level of engagement with a digital intervention is necessary in order to achieve any benefit [22,23], and as such, engagement is often used as a proxy indicator of behavior change or health outcomes. However, engagement measures are not as robust as behavior change or health outcomes (longer engagement might, for example, reflect difficulties in understanding or navigating through the site), and health practitioners and clinicians should collaborate with developers to conduct randomized trials with health outcomes, in order to improve the body of literature on IA.

### Strengths and Limitations

There were some limitations to the review process itself. First, we did not prospectively register our literature review, thereby risking duplication. Also, because search terms attempted to capture websites relating to such a broad topic (ie, health behavior change), it is possible that despite the long list of health terms included, we missed relevant IA and heath behavior change studies.

Despite these limitations, the results of the Crutzen et al study do suggest that less user control (ie, tunnel design) may increase website use and knowledge gained [18]. Less user control may have more impact in a health behavior change context for a variety of reasons [1]. First, a tunnel experience may avoid overwhelming users with too many options by controlling what the user sees and when. In addition, tunnel design can provide a more tailored user experience by tracking users’ progress and knowledge attainment (via tests or assessments) and delivering appropriate content accordingly [1]. Feedback and reinforcement not only personalize the user experience but also help the individual progress through an intervention program and adopt a behavior change [1,9]. The Weymann et al study included some of these tailoring elements; it is certainly possible that this attributed to the higher engagement levels in the intervention group.

### Future Considerations

More research is needed to explore whether tunneling can improve user engagement and knowledge and to understand how it impacts behavior outcomes. Additionally, the studies identified here examine only navigation systems (specifically, tunneling versus organic design), which is just one component of information architecture. Future research should consider the effectiveness of other IA organizing designs (ie, hierarchical and matrix design), as well as other IA elements, such as labeling systems (ie, how information is represented). Some research is beginning to explore the effect of enhanced search systems (ie, how users look for information) within a health-related website [24].

Future reviews might also consider a larger scope of literature. For our purposes, we considered only peer-reviewed RCTs. However, there may be a body of gray literature, albeit less robust, on the subject of IA for Web-based health interventions that could be worth investigating given the lack of evidence found here.

The limited evidence base found in this review demonstrates that IA is a largely unstudied aspect of the health behavior field. If a robust evidence base is established and effective IA designs for health behavior change are identified, the development of Web-based interventions could be streamlined. In addition to improved intervention efficacy, evidence-based IAs could free up resources like time and money to enhance other aspects of the intervention such as graphic design, user experience, marketing, or evaluation. Also, the use of A/B or pre-post testing through automated digital platforms could make building an evidence base more feasible.

### Conclusion

Due to the limited evidence base, few claims can be made about the relationship between IA and health and behavior outcomes. There is support for the effect of tunneling on user engagement and knowledge, but more research is needed to support this claim.

This synthesis of information will provide guidance to practitioners designing websites for health behavior and health outcomes. We hope this serves as a starting point for hypothesis generation to improve empirical evidence on IA and health and behavior outcomes.

## Appendix

Multimedia Appendix 1 Search terms.

## Abbreviations

AC: available cases
IA: information architecture
ITT: intention to treat
RCT: randomized controlled trial
T0, T1, T2, T3: baseline, time 1, time 2, time 3
