# Supplementary material for: Information Architecture of Web-Based Interventions to Improve Health Outcomes: Systematic Review
Source: J Med Internet Res. 2018 Mar 21;20(3):e97. doi: 10.2196/jmir.7867 (PMC5978245; doi:10.2196/jmir.7867)
Supplement: Multimedia Appendix 1 [file jmir_v20i3e97_app1.pdf]

Search Terms

[Website* OR "information architecture" OR "website use" OR "user control" OR "user perception*" OR internet OR "person-based approach*" OR "internet intervention*" OR "web-based" OR "mobile phone*" OR "user centered design" OR "user experience" OR usability OR "persuasive design"] AND ["behavior change" OR "behavioral intervention*" OR "behavioral treatment*" OR adherence OR "health promotion" OR "patient education" OR "self-care" OR "E health" OR mHealth OR "health behavior*" OR "online treatment*" OR "health disparit*" OR "life style behavior*" OR "self-management" OR "health counseling" OR "lifestyle intervention*" OR "health intervention*" OR "hypertension" OR "smoking" OR "heart disease" OR "sexually transmitted disease*" OR "sexually transmitted infection*" OR "sexual health" OR "obesity" OR "physical activity" OR "diet" OR "alcohol" OR "addiction" OR "HIV" OR "stroke" OR "diabetes" OR "cancer*" OR "hepatitis" OR "asthma" OR "chronic obstructive pulmonary disease"]

This is a Multimedia Appendix to a full manuscript published in the J Med Internet Res. For full copyright and citation information see http://dx.doi.org/10.2196/jmir.7867
